# The Relationship between the Val158Met Catechol-o-Methyltransferase (COMT) Polymorphism and Irritable Bowel Syndrome

**DOI:** 10.1371/journal.pone.0018035

**Published:** 2011-03-18

**Authors:** Pontus Karling, Åke Danielsson, Mikael Wikgren, Ingegerd Söderström, Jurgen Del-Favero, Rolf Adolfsson, Karl-Fredrik Norrback

**Affiliations:** 1 Division of Medicine, Department of Public Health and Clinical Medicine, Umeå University, Umeå, Sweden; 2 Division of Psychiatry, Department of Clinical Sciences, Umeå University, Umeå, Sweden; 3 Applied Molecular Genomics Group, Department of Molecular Genetics, VIB, Antwerp, Belgium; 4 University of Antwerp, Antwerp, Belgium; University of Michigan, United States of America

## Abstract

**Background:**

The catechol-O-methyltransferase (COMT) enzyme has a key function in the degradation of catecholamines and a functional polymorphism is val158met. The val/val genotype results in a three to fourfold higher enzymatic activity compared with the met/met genotype, with the val/met genotype exhibiting intermediate activity. Since pain syndromes as well as anxiety and depression are associated to low and high COMT activity respectively and these conditions are all associated with irritable bowel syndrome (IBS) we wanted for the first time to explore the relationship between the polymorphism and IBS.

**Methodology/Principal Findings:**

867 subjects (445 women) representative of the general population and 70 consecutively sampled patients with IBS (61 women) were genotyped for the val158met polymorphism and the IBS patients filled out the Hospital-Anxiety-and-Depression-Scale (HADS) questionnaire, and an IBS symptom diary.

**Results:**

There was a significantly higher occurrence of the val/val genotype in patients compared with controls (30% vs 20%; Chi^2^ (1) 3.98; p = 0.046) and a trend toward a lower occurrence of the val/met genotype in IBS patients compared with controls (39% vs 49%; Chi^2^ (1) 2.89; p = 0.089). Within the IBS patients the val/val carriers exhibited significantly increased bowel frequency (2.6 vs 1.8 stools per day; Chi^2^ (1) 5.3; p = 0.03) and a smaller proportion of stools with incomplete defecation (41% vs 68%; Chi^2^ (1) 4.3; p = 0.04) compared with the rest (val/met+met/met carriers). The val/val carriers also showed a trend for a smaller proportion of hard stools (0% vs 15%; Chi^2^ (1) 3.2; p = 0.08) and a higher frequency of postprandial defecation (26% vs 21%; Chi^2^ (1) 3.0; p = 0.08).

**Conclusions/Significance:**

In this study we found an association between the val/val genotype of the val158met COMT gene and IBS as well as to specific IBS related bowel pattern in IBS patients.

## Introduction

Irritable bowel syndrome (IBS) is a functional gastrointestinal disorder characterized by abdominal discomfort in combination with altered bowel habits in the absence of organic disease. The prevalence of IBS in the western population is estimated to 5–11% [1]. The causes of IBS are not known. Abnormalities in gut immunology, inflammation [2] and brain-gut communications have been proposed [3]. Visceral hypersensitivity is demonstrated in 50–80% of patients with IBS and the origin of this phenomenon is probably based on both peripheral and central nervous system (CNS) mechanisms [1], [4]. Abnormalities in CNS interpretation of visceral afferent signals and dysfunction in the endogenous pain modulation system may contribute to visceral hypersensitivity [3], [5]. Patients with IBS also have high rates of anxiety and depression and patients suffering from anxiety and depression have more IBS-like symptoms [6]–[8]. The pathophysiological links between anxiety and depression and the gut is not fully understood but it has been proposed that changes in the endogenous pain modulation system, the autonomic nervous system (ANS) and the hypothalamus-pituitary-adrenal (HPA) axis may each play a role [3].

Catechol-o-methyltransferase (COMT) has a key function in the degradation of catecholamines (dopamine, noradrenaline and adrenaline). A common polymorphism in the COMT gene (located at chromosome 22q11.2) is val158met (rs4680), which causes a valine (val) substitution to methionine (met) and is responsible for a variation in function of the enzyme. The val/val genotype leads to a three- to fourfold higher activity of the COMT enzyme compared with the met/met genotype, and the val/met genotype shows intermediate activity [9]–[11]. A low COMT activity also results in higher levels of dopamine and chronic activation of dopaminergic neurons which results in lower neuronal content of enkephalin and a decreased activity level of the endogenous pain inhibitory system [9]. The opposite is true with a high COMT activity. Experimental studies in humans (hypertonic saline infusion into the masseter muscle) showed that individuals with the met/met genotype (low COMT activity) exhibited diminished regional μ-opioid receptors activity, higher sensory and affective ratings of pain in response to painful stimuli compared to the val/val individuals [9]. Low COMT activity has also been associated to chronic pain conditions such as facial pain [12], [13], fibromyalgia [14] and with non-migrainous headache [15], whereas the val/val genotype has been associated to anxiety/depression [16].

On the background that both chronic pain syndromes [17] and anxiety/depression [6]–[8] are associated to low and high COMT activity respectively and both are associated with IBS/IBS-like symptoms we wanted for the first time to explore the relationship between the val158met COMT polymorphism and IBS. The hypothesized association between IBS and the COMT polymorphism would based on the aforementioned relationships predict that val/val (high COMT activity) and met/met (low COMT activity) genotypes would be the high risk genotypes. These two genotypes would be associated with increased risk of IBS possibly via separate mechanistic routes whereas the val/met (intermediate COMT activity) genotype would be predicted to be the low risk, protective genotype.

## Methods

### IBS patient and control samples

Seventy consecutive patients with IBS (87% women), all referred from primary care to the gastroenterological out-patient department at the University Hospital of Northern Sweden and all fulfilling the ROME III criteria [18]. The subjects representative of the general population (n = 867, 51% women) were part of a large multiple outcome study, the Betula study, investigating broad domains of cognition, psychiatric and somatic health [8], [19], [20]. Within the Betula study, the participants were recruited by random selection through a population register. The only exclusion criteria for enrollment into the Betula study were dementia, mental retardation, serious visual or auditory handicaps, not having Swedish as a mother tongue or any other feature that would have compromised the ability to comply with the study. None of these conditions or features considered to potentially compromise the ability to comply with the study were exhibited by any of the IBS patients. The representativity of the Betula sample towards the general population of the region of Umeå, Sweden has been demonstrated to be excellent [20]. All subjects within the present study were 50 years or younger, were from the same geographical region of Umeå, Northern Sweden, were Caucasian, had Swedish as their mother tongue and gave their informed (written or verbal) consent to participate. All research in Sweden is required by law to be approved by at least one of six regional committees for human ethics. This study was approved by the ethics committee in Umeå (Regionala Etikprövningsnämden i Umeå). All participants received verbal and written information of the study. All subjects in the Betula project (control group) gave written consent. The IBS patients received written information and gave verbal consent. The IBS subjects were patients at our department, and to avoid to interact the patient-doctor relationship we did not demand a written consent (this was approved by the ethical committee).

### Questionnaires

The patients with IBS prospectively filled in a two week validated symptom diary [21]. The Hospital Anxiety and Depression Scale (HADS), a highly sensitive instrument to screen for symptoms of anxiety and depression among patients with somatic disease, was used to detect these symptoms in the patients with IBS [22], [23]. HADS consists of seven items each for anxiety and depression, each using a 4-point Likert scale (0–3 points). 77% of the IBS patients completed the symptom diary and 97% the HADS questionnaire.

### Medical records

After informed consent from the patients with IBS, records of primary care were searched to define consulters for chronic pain. Consulters for chronic pain were defined as pain more than 6 months, both explained and unexplained pain was included. Ten areas of somatic pain were defined: head, neck, thoracal and lumbar spine, shoulder, arm, hand, hip, knee and foot.

### Val158met COMT

Genotyping was performed by pyrosequencing on a PSQ HS96 pyrosequencer ((Pyrosequencing AB, Uppsala, Sweden). Biotinylated polymerase chain reaction (PCR) products were obtained by performing a 35 cycles PCR reaction (conditions: 30 seconds (sec) at 95 degrees Celsius (°C); 30 sec 68°C ; 30 sec 72°C) using primers: ACCCAGCGGATGGTGGATTT (COMT-F biotinylated) CCTTTTTCCAGGTCTGACAAC (COMT-R). Next, the obtained biotinylated PCR products were immobilized onto streptavidin-coated sepharose beads (Amersham Biosciences, Sweden). Biotinylated single strand DNA was obtained by incubating the immobilized PCR products in 0.5 mol/L NaOH, followed by two sequential washes in 10 mmol/L Tris-acetate, pH 7.6. Primer annealing for SNP analysis (GCACACCTTGTCCTTCA) was performed by incubation at 80°C for 2 minutes and then at room temperature for 5 minutes [24].

### Statistics

We used PASW statistics 17.0.2 (SPSS Inc., Chicago, IL, USA). Median values were used for all ordinal scales, including proportions. Non-parametric Kruskal-Wallis tests were used for comparisons between groups concerning ordinal data. Student t-test was used for continuous data. Chi^2^ test was used for cross table analyses. Logistic regression was used to adjust for age and gender. A p-value less than 0.05 was regarded as statistically significant and a p-value between 0.05 and 0.15 as borderline significant. The results were not corrected for multiple testing. Statistical power calculations (http://www.dssresearch.com/toolkit/spcalc/power.asp) were performed for key analyses.

## Results

### Comparisons concerning the val158met COMT polymorphism genotype frequencies between the IBS patients and the sample representative of the general population

The control subjects were older (mean age 43.5 years vs 31.1 years; p<0.001) and had more male subjects (49% vs 13%, Chi^2^ (1) 33.1; p<0.001) compared to the patients with IBS. There was a higher occurrence of the val/val genotype in patients compared with controls (30% vs 20%; Chi^2^ (1) 3.98; p = 0.046) and a trend toward a lower occurrence of the val/met genotype in IBS patients compared with controls (39% vs 49%; Chi^2^ (1) 2.89; p = 0.089). Comparing the women only we found no significant differences in the genotype frequencies between controls and patients. None of the 9 male IBS patients had the met/met genotype (Table 1). Using logistic regression adjusting for gender and age we found a borderline significant association between IBS and the val/val genotype (adjusted OR: 2.02; CI: 0.95–4.29) and a lower occurrence of the val/met genotype in patients with IBS (adjusted OR: 0.57; CI: 0.28–1.13).

**Table 1:** The distribution of the val158met COMT polymorphism in patients with IBS compared to a sample representative of the general population.

	met/met	val/met	val/val
**IBS women+men (n = 70)**	31%	39%	30%*
**Controls women+men (n = 867)**	31%	49%	20%
**IBS women (n = 61)**	36%	38%	26%
**Controls women (n = 445)**	32%	48%	20%
**IBS men (n = 9)**	0%	44%	56%
**Controls men (n = 422)**	30%	51%	19%

There was a higher occurrence of the val/val genotype in patients compared with controls (30% vs 20%; Chi^2^ (1) 3.98; p = 0.046) and a trend toward a lower occurrence of the val/met genotype in IBS patients compared with controls (39% vs 49%; Chi^2^ (1) 2.89; p = 0.089).

*Statistically significant; p<0.05.

### Gastrointestinal symptoms in patients with IBS in relation to val158met COMT polymorphism

Within the IBS patient sample, the val/val genotype, based on a two week validated symptom diary, was significantly associated with increased bowel frequency (2.6 vs 1.8 stools per day; Chi^2^ (1) 5.3; p = 0.03) and with a smaller proportion of stools with incomplete defecation (41% vs 68%; Chi^2^ (1) 4.3; p = 0.04) compared with the rest (val/met+met/met carriers). There was also a trend towards a smaller proportion of hard stools (0% vs 15%; Chi^2^ (1) 3.2; p = 0.08) and a higher frequency of postprandial defecation (26% vs 21%; Chi^2^ (1) 3.0; p = 0.08) among the val/val carriers compared with the rest (Table 2).

**Table 2:** Characteristics of the patients with IBS in relation to val158met COMT polymorphism based on the symptom diary.

	met/met carriers (n = 19)	val/met carriers (n = 18)	val/val carriers (n = 17)	Chi^2^ (2); All three genotype carriers vs each other p-value	Chi^2^ (1); val/val vs the other carriers; p-value
**Mean pain hours per day**	2.6	2.7	3.5	ns	ns
**Mean bloating hours per day**	3.9	3.5	3.0	ns	ns
**Mean stools per day**	1.9	1.5	2.6	6.5; p = 0.04*	5.3; p = 0.03*
**Percentage of days with no stools**	7%	16%	0%	10.1; p = 0.006*	6.4; p = 0.02*
**Percentage of loose stools**	27%	48%	47%	ns	ns
**Percentage of hard stools**	16%	13%	0%	ns	3.2; p = 0.08
**Percentage of stools with urgency**	52%	38%	42%	ns	ns
**Percentage of stools with straining**	45%	52%	42%	ns	ns
**Percentage of stools with incomplete emptying**	80%	55%	41%	4.4; p = 0.12	4.3; p = 0.04*
**Percentage of meals followed by defecation (Gastro-colon reflex)**	23%	18%	26%	5.5; p = 0.07	3.0; p = 0.08

54 IBS subjects (48 women) completed the symptom diary. The val/val genotype was significantly associated with multiple measures clustering towards IBS-diarrhea-like symptoms compared with the rest (val/met+met/met carriers). Statistics: Kruskal-Wallis (all three genotypes compared against each other in column 5, and val/val carriers compared with the other carriers grouped together in column 6).

*Statistically significant: p<0.05. Borderline statistically significant: p values between 0.05–0.15. ns = non-significant.

### Anxiety, Depression and Health seeking behaviour in relation to the val158met COMT polymorphism

There was no significant difference in HADS scores between different genotypes among the patients with IBS. The val/met carriers tended to have less HADS-depression score compared to the other genotypes (2.5 vs 5.0; Chi^2^ (1) 2.6; p = 0.11). There was no difference in health seeking behavior (visits per year in primary care) between the different genotypes except a trend for more visits per year in primary care for subjects with met/met genotype within the IBS patient sample (2.0 vs 1.4; Chi^2^ (1) 2.7; p = 0.10) (Table 3).

**Table 3:** Anxiety, depression, health seeking behaviour and consulting for chronic pain in relation to the val158met COMT polymorphism among patients with IBS.

	met/met carriers (n = 22)	val/met carriers (n = 27)	val/val carriers (n = 21)	statistics
**Mean age (years)**	32.2	29.7	31.9	ns
**Mean BMI**	24.6	25.2	23.0	ns
**HADS-Anxiety**	9.5	7.5	8.0	ns
**HADS-Depression**	5.0	2.5	5.0	val/met vs others: chi^2^ (1) 2.6; p = 0.11
**Visits per year in primary care**	2.0	1.4	1.7	met/met vs others: chi^2^ (1) 2.7; p = 0.10
**Two or more parts of the body with chronic somatic pain**	45%	27%	38%	ns
**Chronic headache**	23%	18%	19%	ns
**Chronic neck pain**	36%	19%	19%	met/met vs others: chi^2^ (1) 2.5; p = 0.12
**Chronic lumbago**	36%	22%	29%	ns

There was no significant difference in age, BMI, HADS scores, health seeking behavior and consulting for chronic pain between different genotypes among 70 patients (61 women) with IBS. However, the val/met carriers tended to have less HADS-depression score compared to the other genotypes and the met/met genotype tended have more visits per year in primary care and more chronic neck pain. BMI = Body mass index. HADS = Hospital anxiety depression scale. ns = Non-significant.

*Statistically significant: p<0.05. Borderline statistically significant: p values between 0.05–0.15. ns = non-significant.

### Chronic pain in relation to the val158met COMT polymorphism

Consulting behavior for chronic somatic symptoms was analyzed in the patients with IBS in relation to the val158met polymorphism. We found no significant differences in chronic pain (more than two areas of chronic somatic pain), chronic headache, chronic neck pain and chronic lumbago between subjects carrying the three different COMT genotypes. There was a trend towards more consulting for pain in the met/met genotype compared to the other genotypes almost reaching statistical significance for neck pain (36% vs 19%; Chi^2^ (1) 2.5; p = 0.12) (Table 3).

### Statistical Power analyses

Power calculations were performed for key analyses within the study. The statistical power using a one-tail test and an alpha error of 5% concerning the frequency difference of the val/val genotype between IBS patients and controls was 62%. We calculated that the preferable number of IBS patients should have been 127 patients to reach 80% statistical power. The statistical power employing a one-tail test and an alpha error of 5% concerning the frequency difference in the number of stools between IBS patients carrying the val/val genotype compared to the IBS patients carrying the val/met and the met/met genotypes was 72%, with an additional 10 more subjects needed to reach 80% statistical power.

## Discussion

COMT is a key regulator in the degradation of catecholamines and individual differences in the activity of the enzyme have been shown to influence the interpretation of pain and negative environmental stimuli [9], [25]. This study is to our knowledge the first study to explore the relationship between COMT polymorphism and IBS. We found a significantly higher occurrence of the val/val genotype and a trend towards a lower occurrence of the val/met genotype among the patients with IBS compared to the control population from the same geographical region (Umeå, Sweden). These differences were based on comparisons between the total patient and control samples but did not remain significant if only female IBS patients and female controls were included in the analysis. The study was slightly underpowered and since the control sample was representative of the general population the sample is expected to contain subjects suffering from IBS but the diagnostic status with respect to IBS within the control sample was not available. These facts might explain the loss of significance when the women were analyzed separately.

However, using logistic regression, adjusting for age and gender there was a borderline significant association between the val/val genotype and IBS and a trend towards a protective effect of the heterozygous val/met genotype. Patients seeking help for IBS are predominately women and there are also some differences in symptom presentation between men and women, where abdominal pain and constipation-related symptoms are more common in women whereas men more likely report diarrhea-related symptoms [26]. The patients with the val/val genotype in our study (both men and women) reported prospectively more bowel movements, fewer harder stools and more stools after meals than the patients with the other genotypes indicating an association between the val/val genotype and the diarrhea-dominant IBS-subtype.

Both population studies and studies on patient with IBS show a significant correlation between symptom of anxiety and diarrhea [8], [19]. The val/val genotype has been associated with anxiety (Caucasian women) [16], to faster and better recognition of negative facial expression [25], and a reduction in the ability to experience rewards [27]. Anxiety and confrontation with fearful faces have also been shown to enhance perception of signals from the gut [28]–[30] which raises the question whether the val/val genotype predisposes towards increased perception of abnormal gut physiological events. In the present study the patients with IBS showed no differences in HADS-anxiety score between the different genotypes but this does not exclude that the gastrointestinal tract of the “val/val” individuals are more sensitive to activation of the stress system resulting in increased bowel movements and/or lower threshold for defecation signals. In addition, other mechanisms than stress related pathways could be involved, and the influence of an increased degradation of catecholamines (high COMT activity) on the gastrointestinal tract is probably complex and may involve both central and peripheral actions.

A recent Japanese study on patients with functional dyspepsia also showed in consistency with our data a lower frequency of the met allele among dyspeptic patients but the difference was not significant [31].

The met/met genotype with a decreased COMT activity has been associated with a decreased activity level of the endogenous pain inhibitory system and increased chronic somatic pain. For example a low COMT activity has been associated with chronic facial pain [12], [13], fibromyalgia [14] and women with non-migrainous headache [15].

In the present study we found no significant differences in consulting behaviour for chronic pain, but a trend for more chronic pain, especially neck pain in the IBS patient carrying the met/met allele. However within the present study the met/met genotype carriers did not differ in prospectively reporting abdominal pain, in comparison with the other genotype carriers. In a large Norwegian study, investigating musculoskeletal symptoms in a sample representative of the general population they found no association between different musculoskeletal symptoms including chronic chest/abdominal pain and the different val158met COMT genotypes [32]. Neither did they find an association between neuropathic pain and the val158met COMT polymorphism [33]. However, these conflicting data does not exclude that the met/met genotype predisposes to pain in subgroups of patients, for example in individuals with severe multifocal pain. Lastly, there is diversity in the pathophysiological mechanisms of visceral and somatic nociceptive pain [4], so COMT activity may influence the perception of musculoskeletal and abdominal pain differently.

To conclude, in this study we aimed to study the relationship between COMT function and IBS, both of which have been demonstrated to be associated with pain and anxiety/depression. We found a significant association between the val/val genotype and IBS and a trend towards a protective role of the heterozygous val/met genotype for IBS. The val/val genotype was associated with diarrhea-like symptomatology in patients with IBS. We believe that this study justify further research of the val158met COMT polymorphism in a larger samples of IBS patients. It would be warranted and interesting to investigate these genotypes in different subgroups of IBS patients, for example in IBS patients with and without other unexplained pain syndromes, and in IBS patients with different bowel patterns.
